# Genetic analysis of congenital unilateral renal agenesis in children based on next-generation sequencing

**DOI:** 10.1038/s41390-024-03178-4

**Published:** 2024-06-07

**Authors:** Huiru Yang, Jingzhi Zhang, Yao Tang, Qiang Zhong, Wen Qian, Zhengrong Wang, Zunlun Zhou, Zulong Zhang, Wei Pan

**Affiliations:** 1 Department of Nephrology, The Affiliated Hospital of Guizhou Medical University, Guiyang, China; 2 Prenatal Diagnosis Center in Guizhou Province, The Affiliated Hospital of Guizhou Medical University, Guiyang, China; 3 School of Public Health, the key Laboratory of Environmental Pollution Monitoring and Disease Control, Ministry of Education, Guizhou Medical University, Guiyang, China; 4 Department of Obstetrics and Gynecology, The Affiliated Hospital of Guizhou Medical University, Guiyang, China; 5 Department of Gynecology, Guizhou Hospital of The First Affiliated Hospital, Sun Yat-sen University, Guiyang, China

## Abstract

**Background:**

Congenital unilateral renal agenesis (URA) is a kind of rare birth defect during fetal development with varies clinical phenotypes. The pathogenesis and the relationship between gene and phenotype are still unclear.

**Methods:**

Ten URA fetuses were followed up after birth using postnatal renal ultrasound examination to confirm the diagnosis with nine children were URA and one was Renal Ectopy (RE). Trio- WES, CNV- seq were performed with the 10 children and their close relatives.

**Results:**

There were 3 heterozygous variants of *CHD7*, *PROKR2* and *NRIP1* genes were identified in 3 children, respectively. *CHD7* (c.2663T>C, p.M888T) is classified as likely pathogenic (LP), *PROKR2* (c.685G>C, p.G229R) and *NRIP1* (c.2705T>G, p.F902C) are classified as variants of uncertain significance (VUS). *CHD7* (c.2663T>C, p.M888T) and *PROKR2* (c.685G>C, p.G229R) as URA-related genes may be associated with idiopathic hypogonadotropic hypogonadism (IHH) or CHARGE syndrome (CS), and 3D-protein structure prediction revealed that the two variants may affect the stability in the CHD7 protein or PROKR2 protein, separately. The RE-related gene *NRIP1* (c.2705T>G, p.F902C) may be causative of congenital anomalies of the kidneys and urinary tract (CAKUT).

**Conclusions:**

Identification of these variants can in exploring the etiology of URA or RE and improve the level of genetic counseling.

**Impacts:**

- We performed trio-whole-exome sequencing (trio- WES) and copy number variation sequencing (CNV- seq) in 10 children, including 9 children with Unilateral Renal Agenesis and 1 with Renal Ectopy after birth.
- The possible pathogenic genes of URA can be screened using prenatal and postnatal diagnosis of URA fetuses and gene detection after birth.
- Future studies evaluating this association may lead to a better understanding of URA and elucidate exploring the etiology of URA or RE and improve the level of genetic counseling.

## Introduction

Human renal development begins in the first trimester of pregnancy, the three stages of kidney development are the pronephros, mesonephros, and metanephros. The pronephros consists of simple tubules and forms at the 3rd week of gestation, whereas the mesonephros forms at the 4th week. The ureteric bud is the consequence of invasion of the mesonephric duct surrounding the metanephric mesenchyme during the 5th week of gestation, and signaling from the tips of the branching ureteric bud induces nephron formation, with conversion of the metanephric mesenchyme to renal epithelia.^1^ Congenital unilateral renal agenesis (URA), with a prevalence of 2.8/10,000, is a kind of birth defect caused by failure of ureteric bud formation or metanephric mesenchyme mechanism dysfunction during fetal development.^2^ As a frequent form of congenital abnormality, URA can be diagnosed during pregnancy, but clinical phenotypes vary greatly. At present, there are few genetic studies with the URA group in Guizhou Province, where ethnic minorities are abundant. It is thus of great significance to conduct gene sequencing for URA children in Guizhou Province with multi-ethnic groups to find possible pathogenic genes and related diseases. In this study, ten fetuses with URA detected by prenatal ultrasound screening were followed up after birth. Postnatal ultrasound and renal function tests based on blood and urine samples were performed to observe the development of the solitary kidney during growth and development. In addition, trio-whole-exome sequencing (trio-WES) for family members as well as whole-genome copy number variation sequencing (CNV-seq) were carried out to explore possible genetic gene variants. The aims of this study were to provide basic information for genetic analysis, a foundation for prenatal diagnosis, and postnatal clinical management of URA and RE patients.

## Materials and methods

### Patients

Ten fetuses with URA at the Affiliated Hospital of Guizhou Medical University between January 2015 and April 2020 were enrolled. This study was approved by the Ethics Committee of the Affiliated Hospital of Guizhou Medical University (2019-173) and was conducted in accordance with the 1964 Helsinki Declaration and its later amendments. Written informed consent was obtained from the parents of each patient, and 10 mL peripheral blood was collected for genetic analysis and biochemical indicators of renal function; 5 mL urine was collected for routine urine testing.

### Urological ultrasound examination

Ten children who were diagnosed with URA prenatally were reexamined by postnatal urinary ultrasound to determine the specific conditions of the bilateral kidneys after birth. The shape, size and blood flow of the solitary kidney should be observed. Additionally, the immediate family members of the patients, including biological parents and siblings, were examined by urinary ultrasound.

### Detection of renal function indexes using blood and urine

Peripheral blood samples were collected from the 10 children and their family members for biochemical indicators of renal function (creatinine, cystatin C, urea nitrogen); and urine samples were collected for routine urine testing (Urine Protein).

### Karyotype analysis

Heparin-anticoagulated lymphocytes were cultured and harvested after hypotonicity, fixation, dripping, baking and G-banding. Staining results were observed under a light microscope (Olympus, Japan), and the chromosome specimen slide was placed on an automatic chromosome scanning platform (Zeiss, Germany) for image scanning. Twenty phases of karyotypes were counted according to International System of Human Chromosome Nomenclature (ISCN 2016), and 5 karyotypes were analyzed. If abnormal karyotypes were found, an additional 100 karyotypes were analyzed to confirm the result.

### Whole-genome copy number variation sequencing analysis (CNV-seq)

CNV-seq was performed by Berry Genomics, Inc. (Beijing, People’s Republic of China). Bioinformatics analysis was performed as follows. First, 50 ng of DNA was fragmented, and DNA libraries were constructed by end filling, adapter ligation, and PCR amplification. The DNA libraries were subjected to massively parallel sequencing using the NextSeq 500 platform (Illumina, San Diego, CA) to generate approximately 5 million raw sequencing reads with genomic DNA sequences of 36 base pairs in length. The hg19 genomic sequence was used as a reference for map reads. The CNVs identified and mapped were compared against publicly available databases, including Decipher, Database of Genomic Variants (DGV), 1000 Genomes, and Online Mendelian Inheritance in Man (OMIM). Pathogenicity was assessed according to the guidelines outlined by the American College of Medical Genetics (ACMG) for interpretation of sequence variants. Variants were classified as either pathogenic, likely pathogenic, variant of uncertain significance (VUS), likely benign, or benign.

### Trio-whole-exome sequencing (trio-WES) analysis

To identify causal variants for a family, trio or quad familial WES was employed. Gene sequencing and bioinformatic analysis were performed by Berry Genomics, Inc. (Beijing, People’s Republic of China). In brief, genomic DNA was extracted using a Qiagen DNA Blood Midi/Mini kit (Qiagen GmbH, Hilden, Germany), hybridized and enriched. The NovaSeq 6000 platform (Illumina, San Diego), with 150 bp paired-end sequencing mode, was used for sequencing the genomic DNA of each family. The sequencing reads were aligned to the human reference genome (hg38/GRCh38) using the Burrows‒Wheeler Aligner tool. Verita Trekker® Variants Detection System by Berry Genomics and the third-party software GATK (https://software.broadinstitute.org/gatk/) were employed for variant calling. Variant annotation and interpretation were conducted by ANNOVAR.^3^ and Enliven® Variants Annotation Interpretation System authorized by Berry Genomics. The annotation databases include gnomAD (http://gnomad.broadinstitute.org/), the 1000 Genome Project (http://browser.1000genomes.org), SIFT (http://sift.jcvi.org), Mutation Assessor (http://mutationassessor.org), CADD (http://cadd.gs.washington.edu), OMIM (http://www.omim.org), ClinVar (http://www.ncbi.nlm.nih.gov/clinvar), HGMD (http://www.hgmd.org), and HPO (https://hpo.jax.org/app/), among others. The variants were classified into five categories—“pathogenic”, “likely pathogenic”, “uncertain significance”, “likely benign” and “benign”—according to the ACMG guidelines for interpretation of genetic variants.^4^ For trio analysis, potential monogenetic inheritance patterns, including de novo, autosomal recessive, autosomal dominant, X-linked recessive inheritance, mitochondrial, and, where possible, imprinted gene variation, were analyzed. Once a variant was considered to be related to the etiology of a recessive disorder, manual inspection for coverage and additional variants of the entire coding domain was undertaken using Integrated Genomics Viewer. Suspected SNV/Indels were validated using Sanger sequencing.

The three-dimensional (3D) structure of the protein products of missense variants was analyzed by Swiss-Model (http://swissmodel.expasy.org).

## Results

### Ultrasound of the urinary system and renal function indexes of children

We recruited 10 children who were diagnosed prenatally with URA, 6 of whom were ethnic minorities including Miao, Dong, Buyi, and Tujia. The first step was to examine the urinary system of the children and their direct family members and to assess renal function indexes using blood and urine samples. After postnatal ultrasound exzamination, nine children (URA-1-9) were confirmed to have URA; the other child (RE-10) had renal ectopy, with the bilateral renal arteries detected by ultrasound imaging as having a good flow status. In addition, the urinary systems of all family members were normal. The ten children had normal creatinine and urea test results, but cystatin C showed an overall increasing trend. There were no positive results in urine tests (Table 1).

**Table 1:** Clinical information and laboratory examinations of 10 patients with Congenital Unilateral Renal Agenesis (URA) and Renal Ectopy (RE).

Patient No.	Sex	Age in postnatal ultrasound (y)	Nationality	Creatinine (umol/L)	Urea (mmol/L)	Cystatin C (mg/L)	Urine protein (−/+)	Length of solitary kidney (cm)
URA-1	Male	2	Han	3.19	1.65	1.63	−	Left, 8.0
URA-2	Female	2	Han	13.80	3.93	1.48	−	Left, 7.6
URA-3	Female	1	Han	7.23	5.13	1.27	−	Right, 7.9
URA-4	Male	0.5	Buyi	17.44	1.00	1.25	−	Right, 6.9
URA-5	Male	1	Dong	12.91	6.00	1.22	−	Left, 6.5
URA-6	Male	3	Han	4.99	6.68	1.07	−	Left, 9.2
URA-7	Female	0.5	Tujia	15.52	2.11	1.67	−	Left, 5.6
URA-8	Male	1.5	Dong	8.08	6.12	1.10	−	Left, 8.2
URA-9	Female	2	Buyi	0.27	5.03	1.08	−	Left, 8.3
RE-10	Male	5	Miao	31.00	5.20	0.97	−	Left, 7.2；Right, 5.9

### Whole-exome sequencing analysis of the patients

We performed karyotype analysis and CNV-seq analysis on each patient. All karyotypes were 46, XN, and there were no pathogenic CNVs were found (data shown in Supplement 1). We then performed trio-WES on each patient and their family members. A total of 3 variants were identified in 3 of the 10 patients in relation to 3 genes: CHD7 (URA-1), PROKR2 (URA-6) and NRIP1 (RE-10). All variants were missense and in the heterozygous state. One variant in CHD7 was classified as likely pathogenic (LP) according to ACMG guidelines; the other two were classified as VUS (data shown in Table 2).

**Table 2:** Bioinformatic analysis of sequence variants found in 10 patients with Congenital Unilateral Renal Agenesis (URA) and Renal Ectopy (RE) using trio-whole-exome sequencing.

Patient No.	Zygosity	Gene	Location	Subre-gion	Variant	Mutation type	Amino acid change	Origin of the mutation	ACMG
URA-1	Hetero	*CHD7*	Chr8:60820056-60820056	Exon9	c.2663T>C	Missense	p.M888T	De novo	LP:PS2 + PM1 + PM2 + PP2
URA-2	No pathogenic Single nucleotide variation (SNV) and Insertion-deletion (InDel) variants related to URA of this case were detected.								
URA-3	No pathogenic SNV and InDel variants related to URA of this case were detected.								
URA-4	No pathogenic SNV and InDel variants related to URA of this case were detected.								
URA-5	No pathogenic SNV and InDel variants related to URA of this case were detected.								
URA-6	Hetero	*PROKR2*	Chr20:5302510-5302510	Exon3	c.685G>C	Missense	p.G229R	inherited from father	VUS:PM2 + PS3_Supporting
URA-7	No pathogenic SNV and InDel variants related to URA of this case were detected.								
URA-8	No pathogenic SNV and InDel variants related to URA of this case were detected.								
URA-9	No pathogenic SNV and InDel variants related to URA of this case were detected.								
RE-10	Hetero	*NRIP1*	chr21:14965488-14965488	Exon4	c.2705T>G	Missense	p.F902C	inherited from mother	VUS:PM1 + PM2

*ACMG* American College of Medical Genetics and Genomics guidelines, *VUS* variant of uncertain significance, *LP* likely pathogenic variant.

#### CHD7

**Fig. 1:**
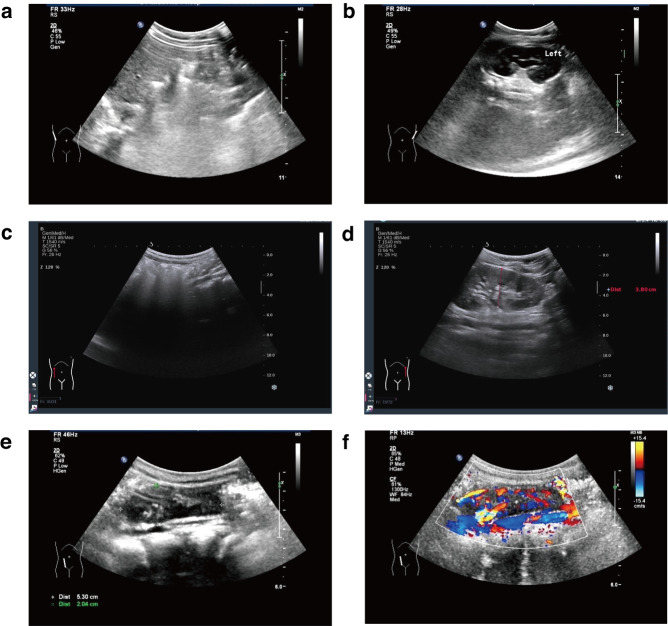
Postnatal ultrasound screening of patients URA-1, URA-6 and RE-10. **a** Images of patient URA-1 with a diagnosis of right renal agenesis. **b** By ultrasound, the left kidney of patient URA-1 can be seen, with mild hydronephrosis. **c** By ultrasound, patient URA-6 was diagnosed with right renal agenesis, as shown in **c**. **d** The left kidney of patient URA-6 can be seen in **d**. **e** Patient RE-10 with a diagnosis of right renal ectopy. On ultrasound, right renal echoes were seen in the right iliac fossa, as shown in **e**. **f** The blood flow signal of the right kidney of patient RE-10 was normal, as indicated in **f**.

**Fig. 2:**
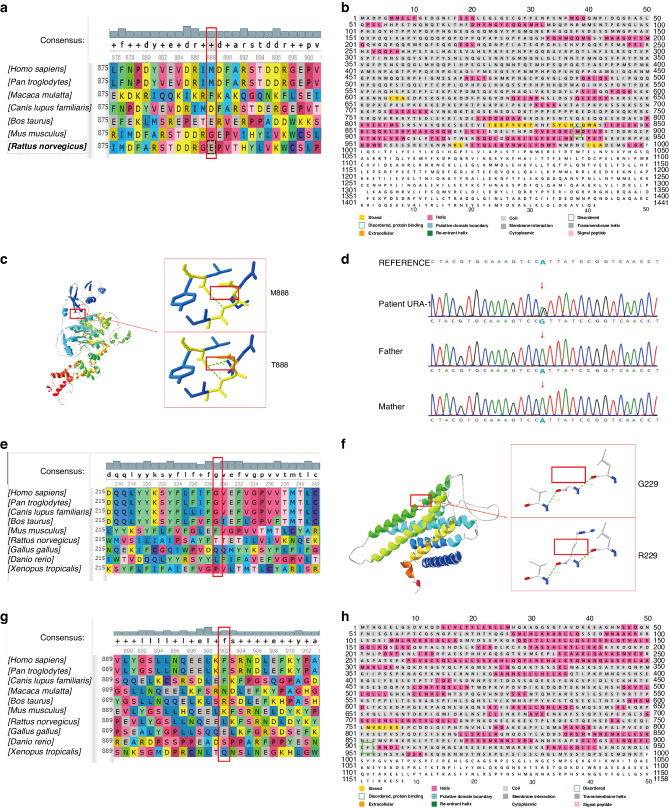
Evaluation of the effects of the CHD7, PROKR2 and NRIP1 gene variation on gene function. Gene variants in patients URA-1 (**a**–**d**), URA-6 (**e**, **f**) and RE-10 (**g**, **h**) alter amino acid conformation. **a** CHD7 M888 is not conserved. **b** M888 is located in the α-helix. **c** 3D models show amino acids of p.M888T forming a hydrogen bond (green dotted line) compared with wildtype. **d** Sanger sequencing validated the CHD7 variant in patient URA-1. The red arrow indicates the variant site c.2663T>C. **e** PROKR2 G229 is conserved in the four species with the greatest sequence similarity. **f** 3D models show that hydrogen bonds of p.G229R do not change, though the side chain does change compared with wildtype. **g** NRIP1 F902 is conserved in species. **h** F902 of NRIP1 is located in the random coil region.

We identified a missense CHD7 variant, c.2663T>C (p.M888T), in one URA child, which was confirmed by Sanger sequencing (Fig. 2d). The patient URA-1 was diagnosed with URA with left renal agenesis and hydronephrosis of the right kidney before and after birth, as examined by ultrasound exzamination (Table 1 and Fig. 1a, b). Blood samples from the parents of patient URA-1 were available. As the results of Sanger sequencing suggested that the parents did not carry the same variant (Fig. 2d), the CHD7 variant c.2663T>C (p.M888T) was de novo. This variant was not found in gnomAD, the 1000 Genome Project (1000 G), the Exome Aggregation Consortium (ExAC), or the Berrybig data population database; it occurs in the chromo functional domain of CD2_tandem_CHD5-9_like of exon 9. This missense mutation of CHD7 is common and may cause phenotypes of related diseases. The proportion of benign mutations is low, and this variant is hence classified as LP (PS2 + PM1 + PM2 + PP2) according to ACMG guidelines. This novel variant has not been reported in the literature.

#### PROKR2

We identified a missense variant PROKR2 variant, c.685G>C (p.G229R), in one URA patient. Patient URA-6 was diagnosed with URA with left renal agenesis before and after birth, as examined by ultrasound (Table 1 and Fig. 1c, d). Blood samples from the parents and sister of URA-6 were available. We found that only his father carried a PROKR2 mutation: the c.685G>C variant. His mother and elder sister did not carry the mutation. This variant was not found in the gnomAD, 1000 G, ExAC, and Berrybig data population databases. It is rated as a disease-causing mutation (DM) in Human Genome Mutation Database (HGMD) and LP in the ClinVar database. Therefore, this variant is classified as VUS (PM2 + PS3_Supporting) according to ACMG guidelines.

#### NRIP1

We identified a missense NRIP1 variant, c.2705T>G (p.F902C), in a patient with renal ectopy (Table 2). The patient RE-10 was diagnosed with URA by prenatal ultrasound, but renal ectopy after birth was assessed with ultrasound (Table 1 and Fig. 1e, f). Blood samples from the parents of RE-10 were subjected to Sanger sequencing, which confirmed that his mother carried the same c.2705T>G variant. The allele frequencies of the c.2705T>G variant in the Berrybig data population database, ExAC, 1000 G and gnomAD are 0.00111145, 0.000189543776371308, 0.000199681 and 0.00250109, respectively (Table 2). This gene encodes nuclear receptor-interacting protein 1, and the mutation occurs in the functional domain of the repression domain in exon 4. Therefore, this variant is classified as VUS (PM1 + PM2) according to ACMG guidelines. This novel variant has not been reported in the literature.

### Structure‒function correlations of variants

#### CHD7

The impact of this missense c.2663T>C variant was evaluated according to the 3D structure of the protein. As shown in Fig. 2, p.M888T variant is located in the α-helix of CHD7 (Fig. 2b). When Met is replaced by Thr, one hydrogen bond would be gained, though the protein structure and stability might be changed (Fig. 2c).

#### PROKR2

The impact of the missense variant c.685G>C was also evaluated. Sequence alignment of the PROKR2 protein among multiple species showed that p.G229 is highly conserved (Fig. 2e), suggesting that G229 may play a vital role in maintaining the stability and function of the protein; indeed, it is conserved in the four species with the highest PROKR2 protein similarity (Fig. 2e). Despite no change in hydrogen bonding, the amino acid side chain is altered; whether it impacts the structure and stability of the protein is unclear (Fig. 2f).

#### NRIP1

The impact of this missense variant c.2705T>G was evaluated. Figure 2g illustrates the conservation of F902 in the three species with the highest similarity. The variant is located in the random coil of NRIP1 (Fig. 2h).

## Discussion

Unilateral renal agenesis occurs when the ureteral bud fails to form the ureter, renal pelvis and renal interstitium.^1^ Renal development begins at the fifth week of gestation and fetal kidneys can be detected using prenatal ultrasound screening during the 9th–12th weeks of pregnancy. Postpartum ultrasound, renal radionuclide imaging or magnetic resonance imaging may further identify URA, renal ectopy or multicystic dysplastic kidney (MCDK).^5^ Most children with URA have a good short-term prognosis, and the first 10 years after birth are similar to those of healthy children. Therefore, some researchers believe that URA might be a harmless congenital condition.^6^ However, some studies have shown that approximately 40–50% of URA patients require dialysis before the age of 30 years.^7^ Gene plays an important role in the developmental process of embryonic kidney. In 2016, more than eighty URA patients were recruited in a research for screening pathogenic genes, 42 pathogenic mutations in 14 candidate genes were found, including SALL1, EYA1, SIX5, et al, but the mutation spectrum was scattered and no hotspot mutations were found.^8^ At present, the suspected pathogenic genes reported include WT1, EYA1, SIX1, and LIM1, with most playing an important role in the signal transduction of kidney development.^9^ Therefore, studies involving broad populations are needed.

In this study, trio-WES and CNV-seq were used to examine genetic pathogenic factors of URA and RE in multi-ethnic children of Guizhou Province. We identified 2 variants in URA children that may be related to idiopathic hypogonadotropic hypogonadism (IHH) or CHARGE syndrome (CS), and 1 variant associated with RE may be related to congenital anomalies of the kidneys and urinary tract (CAKUT). On the basis of previous studies, these genes are thought to be related to renal agenesis. Below, we discuss these genes individually.

### CHD7

CHD7 encodes chromodomain helicase DNA-binding protein 7 and is located at chromosome 8q12.1. CHD7 contains multiple domains, belongs to the ATP-dependent chromatin helicase DNA-binding protein family, and collaborates with other molecules to play a role in chromatin remodeling.^10^ CHD7 is highly expressed in cells of the hypothalamus, olfactory bulb and anterior pituitary during the embryonic period, and plays an important role in the development of the reproductive and nervous system, ear, heart, and other organs. Mutations in CHD7 may cause dysfunction of gonadotropin-releasing hormone (GnRH) neurons and maldevelopment of the olfactory bulb, leading to the inability of the pituitary to secrete sufficient gonadotropin, which can lead to IHH.^11^ In addition, CHD7 mutations may cause CS. CS is an autosomal dominant (AD) disease with an incidence of 1/15000–1/17,000. The clinical features including heart disease, retarded growth, genital agenesis, et al., and clinical penetrance vary widely.^12^ Heterozygous variants in CHD7 have been found to be associated with IHH or CS.^13–16^ The mutation detected in our study is not included at the CHARGE Syndrome mutation website (www.CHD7.org), and no relevant literature reports were found. In our study, 3D protein structure prediction showed that the c.2663T>C variant leads to an additional more hydrogen bond, which may change the structure and stability of the CHD7 protein. The patient URA-1 was 2 years old; levels of sex hormones (luteinizing hormone, follicle-stimulating hormone, prolactin, testosterone, and inhibin B) were normal, and growth curve wasn’t differ from peers. As the patient was young, secondary sexual characteristics had not yet developed, and diagnosis was difficult. It is necessary to follow-up regularly.

### PROKR2

The PROKR2 gene variant c.685G>C (p.G229R), which may cause IHH, was detected in URA-6. IHH is a complex clinical and genetic heterogeneous disease; the overall incidence of IHH is 1–10/100,000.^17^ Recent studies have shown that rare mutations in different genes can cause GnRH neuron migration or dysfunction and lead to IHH, and some pathogenic genes like KAL1, FGFR1, PROK2 and CHD7 were been studied widely.^18^ WANG et al. reported that the mutation rate of PROKR2 was reported to be 18.1% for IHH children.^19^ Mao described an adult male patient diagnosed with IHH carrying the same PROKR2 mutation with small testes but no occurs renal agenesis.^20^ In our study, URA-6 inherited the PROKR2 mutation from his father, though his father had normal reproductive function. According to the 3D predicted protein model, there is no change in hydrogen bonding, but the PROKR2 protein conformation may be changed, which might impact its stability. At present, it cannot be accurately determined whether PROKR2 p.G229R is a pathogenic variant related to IHH or URA, as the children are young, but we will continue to follow the growth of the endocrine system and gonads of this child.

### NRIP1

Nuclear receptor-interacting protein 1 (NRIP1) is a nuclear receptor transcriptional cofactor that acts as a transcriptional repressor. It has been confirmed that NRIP1 gene loss-of-function is a new single-gene mutation cause of human autosomal dominant disease. ZHENG et al. identified a heterozygous frameshift variant of NRIP1 (p. Asn 676 Lys) in a patient diagnosed with CAKUT, including bilateral hydronephrosis and right grade V vesicoureteral reflux (VUR), and his father had left renal agenesis.^21^ The disease spectrum of CAKUT is broad, including renal ectopy, renal dysplasia, hydronephrosis, VUR, etc. Vivante et al. reported that a heterozygous truncation mutation of NRIP1 (c.279delG) causes CAKUT by interfering with retinoic acid transcriptional signaling through dominant mutations, it suggested that dominant NRIP1 mutations may be a causative factor in CAKUT.^22^ In our research, NRIP1 (c.2705T>G, p.F902C) gene mutation was detected in patient RE-10 with renal ectopy after birth, which was inherited from his completely normal phenotypic mother. We hypothesize that this mutation might lead to the appearance of renal ectopy with CAKUT during kidney development, with incomplete penetrance.

In this study, we recruited 10 patients in our research over approximately 5 years and performed genetic analysis on these patients and their families. However, the disadvantage of this study is that the number of patients was insufficient. In the future, we will continue to recruit more children with URA and RE to further explore the genetic pathogenic factors of renal agenesis. At the same time, we need to conduct functional experiments to elucidate the underlying mechanisms of LP and VUS mutations.

In summary, this research demonstrates that URA-related and RE-related genes, including CHD7 (c.2663T>C), PROKR2 (c.685G>C) and NRIP1 (c.2705T>G), may be associated with IHH, CS or CAKUT. As a useful high technology, trio-WES can be used for genetic disease detection focusing on the development monitoring of children, and timely intervention and treatment can be carried out. The outcome may be beneficial for prenatal diagnosis and prognostic management.

## Supplementary information

Supplement 1

## Data Availability

All data generated or analyzed during this study are included in this article and its supplementary material files. Further enquiries can be directed to the corresponding author.

## References

[1] Rosenblum, S., Pal, A. & Reidy, K. Renal Development in the Fetus and Premature Infant. *Semin. Fetal Neonatal Med.* **22**, 58–66 (2017). 28161315 10.1016/j.siny.2017.01.001 PMC5387761

[2] Laurichesse Delmas, H. et al. Congenital Unilateral Renal Agenesis: Prevalence, Prenatal Diagnosis, Associated Anomalies. Data from Two Birth-Defect Registries. *Birth Defects Res.* **109**, 1204–1211 (2017). 28722320 10.1002/bdr2.1065

[3] Wang, K., Li, M. & Hakonarson, H. Annovar: Functional Annotation of Genetic Variants from High-Throughput Sequencing Data. *Nucleic Acids Res.* **38**, e164 (2010). 20601685 10.1093/nar/gkq603 PMC2938201

[4] Richards, S. et al. Standards and Guidelines for the Interpretation of Sequence Variants: A Joint Consensus Recommendation of the American College of Medical Genetics and Genomics and the Association for Molecular Pathology. *Genet. Med.* **17**, 405–424 (2015). 25741868 10.1038/gim.2015.30 PMC4544753

[5] Westland, R., Schreuder, M., Ket, J. & van Wijk, J. Unilateral Renal Agenesis: A Systematic Review on Associated Anomalies and Renal Injury. *Nephrol. Dial. Transplant.* **28**, 1844–1855 (2013). 23449343 10.1093/ndt/gft012

[6] La Scola, C. et al. Congenital Solitary Kidney in Children: Size Matters. *J. Urol.* **196**, 1250–1256 (2016). 27060778 10.1016/j.juro.2016.03.173

[7] Sanna-Cherchi, S. et al. Renal Outcome in Patients with Congenital Anomalies of the Kidney and Urinary Tract. *Kidney Int.* **76**, 528–533 (2009). 19536081 10.1038/ki.2009.220

[8] Wu, H. et al. Identification of 8 Novel Mutations in Nephrogenesis-Related Genes in Chinese Han Patients with Unilateral Renal Agenesis. *Am. J. Nephrol.* **46**, 55–63 (2017). 28618409 10.1159/000477590

[9] Sanna-Cherchi, S. et al. Genetic Approaches to Human Renal Agenesis/Hypoplasia and Dysplasia. *Pediatr. Nephrol.* **22**, 1675–1684 (2007). 17437132 10.1007/s00467-007-0479-1 PMC1994209

[10] Hale, C., Niederriter, A., Green, G. & Martin, D. Atypical Phenotypes Associated with Pathogenic Chd7 Variants and a Proposal for Broadening Charge Syndrome Clinical Diagnostic Criteria. *Am. J. Med. Genet. Part A* **170**, 3367–3368 (2016). 26996150 10.1002/ajmg.a.37629

[11] Balasubramanian, R. & Crowley, W. Reproductive Endocrine Phenotypes Relating to Chd7 Mutations in Humans. *Am. J. Med. Genet. Part C. Semin. Med. Genet.* **175**, 507–515 (2017). 29152903 10.1002/ajmg.c.31585 PMC5790312

[12] Usman, N. & Sur, M. CHARGE Syndrome. *In: StatPearls [Internet].* (StatPearls Publishing, Treasure Island (FL), 2022).

[13] van Ravenswaaij-Arts, C. & Martin, D. New Insights and Advances in Charge Syndrome: Diagnosis, Etiologies, Treatments, and Research Discoveries. *Am. J. Med. Genet. Part C. Semin. Med. Genet.* **175**, 397–406 (2017). 29171162 10.1002/ajmg.c.31592 PMC6591023

[14] Li, J. et al. Phenotypic Spectrum of Idiopathic Hypogonadotropic Hypogonadism Patients with Chd7 Variants from a Large Chinese Cohort. *J. Clin. Endocrinol. Metab.* **105**, dgz182 (2020). 31689711 10.1210/clinem/dgz182

[15] Siavrienė, E. et al. A Novel Chd7 Variant Disrupting Acceptor Splice Site in a Patient with Mild Features of Charge Syndrome: A Case Report. *BMC Med. Genet.* **20**, 127 (2019). 31315586 10.1186/s12881-019-0859-y PMC6637606

[16] Xie, Y., Zheng, R., Han, B., Yuan, H. & Hu, Z. Analysis of Chd7 Gene Variants in 22 Patients with Idiopathic Hypogonadotropic Hypogonadism. *Chin. J. Med. Genet* **39**, 571–575 (2022). 10.3760/cma.j.cn511374-20210415-00331 35773757

[17] Mao, J., Wu, X. & Dou, J. Expert Consensus on the Diagnosis and Treatment of Idiopathic Hypogonadotropic Hypogonadism. *Chin. J. Inter Med.* **54**, 739–744 (2015).

[18] Liu, M. & Wu, L. Advances in Genetic Research of Kallmann Syndrome. *Int. J. Reprod. Health Fam. Plan.* **33**, 186–190 (2014).

[19] Wang, Y., Qin, M., Fan, L. & Gong, C. Correlation Analysis of Genotypes and Phenotypes in Chinese Male Pediatric Patients with Congenital Hypogonadotropic Hypogonadism. *Front. Endocrinol.* **13**, 846801 (2022). 10.3389/fendo.2022.846801 PMC9164197 35669683

[20] Mao, J. Influence of Gene Mutations on Hypothalamus Pituitary Gonad Axis Function and Gonadotropin Induced Spermatogenesis in Male Patients with Idiopathic Hypogonadotropic Hypogonadism. *Chinese Academy of Medical Sciences & Peking Union Medical College.* (2013).

[21] Zheng, B. et al. A Truncating Nrip1 Variant in an Arabic Family with Congenital Anomalies of the Kidneys and Urinary Tract. *Am. J. Med. Genet. Part A* **188**, 310–313 (2022). 34525250 10.1002/ajmg.a.62502 PMC10067133

[22] Vivante, A. et al. A Dominant Mutation in Nuclear Receptor Interacting Protein 1 Causes Urinary Tract Malformations Dysregulation of Retinoic Acid Signaling. *J. Am. Soc. Nephrol.* **28**, 2364–2376 (2017). 28381549 10.1681/ASN.2016060694 PMC5533226

