# Supplementary material for: Genetic analysis of congenital unilateral renal agenesis in children based on next-generation sequencing
Source: Pediatr Res. 2024 Jun 7;97(1):273–9. doi: 10.1038/s41390-024-03178-4 (PMC11798820; doi:10.1038/s41390-024-03178-4)
Supplement: Supplementary file 1 — Supplement 1 [file 41390_2024_3178_MOESM1_ESM.pdf]

SUPPLEMENT 1

Patient No.	CNV-seq analysis	Fragment size	ACMG
URA-1	No chromosome aneuploidy or copy number variants (CNVs) at least 100Kb known to be pathogenic were detected.	——	——
URA-2	No chromosome aneuploidy or CNVs at least 100Kb known to be pathogenic were detected.	——	——
URA-3	seq[hg19]dup(8)(q23.3) chr8:g.112120000_112340000dup.	0.22Mb	VUS
URA-4	No chromosome aneuploidy or CNVs at least 100Kb known to be pathogenic were detected.	——	——
URA-5	seq[hg19]dup(12)(p13.31) chr12:g.8000000_8140000dup.	0.14Mb	Likely Benign(LB)
URA-6	seq[hg19]dup(7)(q11.21) chr7:g.63500000_63820000dup.	0.32Mb	VUS
URA-7	seq[hg19]del(14)(q11.2) chr14:g.22640000_22960000del.	0.32Mb	Benign
URA-8	seq[hg19]dup(2)(q14.1) Chr2:g.116540000_116700000dup； seq[hg19]del(14)(q11.2) chr14:g.22640000_22960000del.	0.16Mb； 0.40Mb	LB； Benign
URA-9	No chromosome aneuploidy or CNVs at least 100Kb known to be pathogenic were detected.	——	——
RE-10	seq[hg19]dup(4)(q21.1q21.1) chr4:g.78060000_78640000dup； seq[hg19]dup(7)(p22.2p22.2) chr7:g.2940000_3320000dup.	0.58Mb； 0.38Mb	VUS
